# Sexual Dimorphism in Mouse Meiosis

**DOI:** 10.3389/fcell.2021.670599

**Published:** 2021-05-10

**Authors:** Rong Hua, Mingxi Liu

**Affiliations:** State Key Laboratory of Reproductive Medicine, Department of Histology and Embryology, School of Basic Medical Sciences, Nanjing Medical University, Nanjing, China

**Keywords:** meiosis, sexual dimorphism, knockout mice, spermatocyte, oocyte

## Abstract

Meiosis is a highly conserved and essential process in gametogenesis in sexually reproducing organisms. However, there are substantial sex-specific differences within individual species with respect to meiosis-related chromatin reorganization, recombination, and tolerance for meiotic defects. A wide range of murine models have been developed over the past two decades to study the complex regulatory processes governing mammalian meiosis. The present review article thus provides a comprehensive overview of the knockout mice that have been employed to study meiosis, with a particular focus on gene- and gametogenesis-related sexual dimorphism observed in these model animals. In so doing, we aim to provide a firm foundation for the future study of sex-specific differences in meiosis at the molecular level.

## Introduction

For mammalian species, life begins when haploid egg and sperm cells fuse with one another. These two types of gametes are generated from diploid precursor cells through tightly regulated processes including an initial round of DNA replication and two subsequent rounds of cellular division (meiosis I and II). Meiosis is a specialized form of cell division that occurs only in the context of gametogenesis wherein diploid cells give rise to genetically diversified haploid progeny (Bolcun-Filas and Handel, 2018). During the initial meiotic prophase I, sister chromatids undergo proteinaceous axial element (AE) structural organization along the chromosome axis to facilitate synaptonemal complex (SC) assembly (Zickler and Kleckner, 1999), after which homologous chromosome pairing (Gerton and Hawley, 2005; Barzel and Kupiec, 2008; Bhalla and Dernburg, 2008), synapsis (Cahoon and Hawley, 2016), and meiotic recombination occur to generate physical chiasmatic linkages between paired chromosomes (Handel and Schimenti, 2010; Baudat et al., 2013; Keeney et al., 2014; Lam and Keeney, 2014). These processes are subject to spatiotemporal regulation by telomeres anchored on the nuclear membrane (Ding et al., 2007). The end result of this process is the physical connection of homologous chromosomes by chiasmata that aid in subsequent chromosomal capture by microtubules from the opposing cellular poles during subsequent metaphase I (MI) (Sakuno et al., 2011). The chromosomes are then separated from one another during anaphase I, segregating to opposite cellular poles following chiasmatic dissolution (Buonomo et al., 2000; Kudo et al., 2006), thereby reducing the number of chromosomes by half in the daughter cells generated at the end of meiosis I (Watanabe, 2012).

While these processes are highly conserved, they also differ significantly in the context of male and female gametogenesis, a phenomenon known as sexual dimorphism in the mammalian meiosis (Hunt and Hassold, 2002). Such sexual dimorphism in meiosis has been studied in many previous reports, including a review prepared by Handel et al. highlighting sexual dimorphism as it pertains to the regulation of cell cycle progression and kinetics during meiosis (Handel and Eppig, 1998). Mammalian oocytes undergo synchronous meiosis during fetal development, followed by two rounds of arrest and resumption of these meiotic processes. In contrast, meiosis in males is a continuous process that occurs in waves throughout adulthood, with new germ cell populations being regularly recruited to undergo meiosis (Handel and Eppig, 1998). Sex chromosomes also impact patterns of meiosis-related transcriptional activity in males and females. In females, sex chromosomes undergo normal recombination and transcription, whereas meiotic sex chromosome inactivation (MSCI) occurs in males in the context of meiosis, as has been reviewed previously (Turner, 2015).

Sexually dimorphic regulatory processes also manifest during mammalian gametogenesis. For example, Morelli et al. have previously reviewed sex-specific differences in the processes of recombination, double-strand break (DSB) repair, and synapsis (Morelli and Cohen, 2005), detailing essential proteins known to be associated with these processes. Many recent advances in gene-editing technologies, however, have led to important breakthroughs in the study of meiotic processes in recent years. The present review was therefore formulated by searching the Pubmed database using “meiosis” and “knockout” as keywords to identify over 700 articles which were then surveyed to identify all proteins that play functionally important roles during meiosis (Supplementary Table 1). While many of these proteins have been studied in detail, mice in which others have been knocked out exhibit sex-specific differences in meiotic phenotypes that have not been discussed at length in prior reviews. By surveying these proteins and associated murine knockout model systems, we herein seek to provide novel insight into the sexual dimorphism of meiosis in order to guide future research efforts.

## The Onset of Meiotic Prophase

**FIGURE 1:**
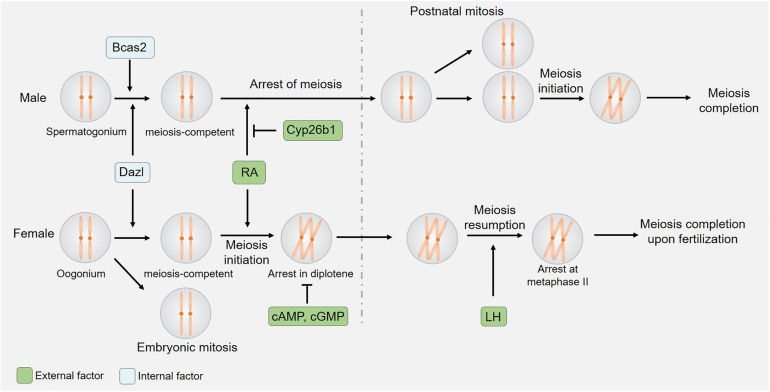
Sexual dimorphism in the initiation and kinetics of meiosis. Both germ cell-derived internal factors and somatic cell-derived external factors regulate meiotic initiation. The expression of CYP26B1 by somatic cells of the embryonic testis suppresses RA-induced meiotic entry, while cAMP/cGMP and LH control oocyte-specific meiotic arrest and resumption.

The onset and regulation of meiosis differ significantly between male and female mice (Figure 1; Handel and Eppig, 1998). Oocyte meiosis begins by embryonic day 13 in these animals, whereas testicular germ cell division at this same time point exhibits stagnation in the mitotic phase. Factors that ultimately govern these sexually dimorphic meiotic initiation phenotypes include retinoic acid (RA) inhibition in male germ cells and the regulation of germ cell entry into a meiosis-competent state.

Both germ cell-intrinsic factors and somatic cell-derived external factors ultimately influence meiotic initiation in mice. In order to enter into meiosis, germ cells must first enter a meiosis-competent state. This state, in turn, is regulated by the *Dazl* gene (Nicholls et al., 2019), which encodes a highly conserved RNA-binding protein that is specifically expressed within germ cells (Seligman and Page, 1998). In murine embryos lacking DAZL expression, primordial germ cells (PGCs) arrive at the gonad but do not undergo appropriate developmental differentiation, instead proliferating and persisting in their expression of pluripotency factors without initiating spermatogenesis or oogenesis at the proper time points (Lin et al., 2008; Chen et al., 2014; Nicholls et al., 2019). While the loss of DAZL interferes with meiosis in both male and female mice, the effects of knocking out this protein were sex-specific. The loss of DAZL disrupted oogenesis prior to the onset of meiosis, whereas spermatogenesis was arrested during the G0 phase of meiosis, with limited SYCP3 expression being evident at this time point (Lin et al., 2008). This suggests that different regulatory mechanisms ultimately govern the entry of male and female germ cells into a meiosis-competent state. Prior work has shown BCAS2 to function as a post-transcriptional regulator of spermatocyte meiosis initiation such that the conditional knockout of BCAS2 in male germ cells results in impaired spermatogenesis and male infertility. While spermatagonia in these *Bcas2* conditional knockout mice appear normal, they rarely enter meiosis prophase I or subsequent phases of meiosis (Liu et al., 2017). The alternative splicing of 245 genes, including *Dazl*, has also been detected in *Bcas2* -null testes. Despite its central role in spermatocyte meiosis, BCAS2 is dispensable in the context of oocyte meiosis (Liu et al., 2017).

The vitamin A derivative RA is produced by somatic cells in the kidneys and gonads, and functions as an important external regulator of meiotic initiation (van Pelt and de Rooij, 1991; Anderson et al., 2008; Endo et al., 2017), serving as a key inducer of meiosis in many contexts (Bowles et al., 2006; Koubova et al., 2006). Stra8 is a protein that is upregulated in response to RA (Baltus et al., 2006), and that is expressed at high levels in germ cells immediately following the initiation of meiosis only to be rapidly downregulated during subsequent stages of this process. As a transcriptional regulator, STRA8 can bind to the promoter regions of thousands of different genes including those associated with meiotic prophase I, the G1-S cell cycle transition, and mitotic program inhibition thereby regulating their expression to orchestrate appropriate meiotic cell division (Kojima et al., 2019). While both spermatogonial cells and oocytes are exposed to RA during embryonic development, such exposure is not sufficient to initiate meiosis in male germ cells. This is believed to be due to the presence of certain meiosis-preventing factors within the fetal and neonatal testis, and experimental studies using testicular extracts and organ cultures lend credence to such a hypothesis (Moor and Warnes, 1979; Gondos et al., 1996). Indeed, recent work has shown that CYP26B1, which is expressed in the fetal testes, can degrade RA and thereby prevent STRA8 upregulation and consequent meiotic initiation (Koubova et al., 2006). The expression of CYP26B1 has been detected in developing seminiferous cord tissues (Bowles et al., 2006; Koubova et al., 2006; Sedlmeier et al., 2014), and *Cyp26b1*-deficient embryos exhibit ectopic STRA8 expression and meiotic initiation within germ cells in the fetal testis (Bowles et al., 2006; MacLean et al., 2007; Sedlmeier et al., 2014). These data thus suggest that CYP26B1-expressing cells serve as a barrier to the exogenous RA-mediated initiation of germ cell meiosis in male mice.

The highly conserved DMRT family of transcription factors harbor the unique DM domain DNA binding motif, and serve as key regulators of meiotic initiation. Specifically, DMRT1 can simultaneously suppress the initiation of meiosis while promoting mitotic proliferation, as evidenced by the results of chromatin immunoprecipitation (ChIP) and transcriptomic studies of mutant cells demonstrating the ability of this transcription factor to regulate STRA8 and CYP26B1 expression (Matson et al., 2010). The expression of DMRT6 is also evident in type A, intermediate, and type B differentiating spermatogonia wherein it coordinates the differentiation of these cells by orchestrating the appropriate transition from the mitotic spermatogonial program to a meiotic spermatocytic program (Zhang et al., 2016). While the DNA binding characteristics and genetic targets of DMRT6 are very similar to those of DMRT1, these two transcription factors have opposing impacts on gene expression. While DMRT1 can promote spermatogonial division and differentiation, DMRT6 instead functions by terminating mitotic division and promoting meiotic entry (Zhang et al., 2014). Notably, DMRT1/6 function as regulators of these meiotic processes specifically in testicular tissues (Zhang and Zarkower, 2017), underscoring the sexually dimorphic regulatory roles of these transcription factors in the context of murine gametogenesis.

Both internal and external factors ultimately regulate germ cell entry into meiosis. The observed sex-specific differences in *Dazl*-knockout-related phenotypes and in the testicular-specific expression of BCAS2 underscore the sexually dimorphic regulation of germ cell entry into a meiosis-competent state. Somatic cell-derived RA, in contrast, serves as an external factor that promotes meiotic entry, whereas DMRT1/CYP26B1 suppress these RA-induced effects in spermatogonia but not oocytes, thereby disrupting the initiation of meiosis in a sex-specific manner (Figure 1).

## Oocyte Meiosis Arrest and Resumption During Prophase I

Spermatocyte meiosis is a continuous and ongoing process in mammals, but the same is not true for oocytes, which undergo initial meiotic progression during embryonic development, only to arrest at the diplotene stage of prophase I (Figure 1). Such arrest persists until immediately prior to ovulation, at which time a preovulatory luteinizing hormone (LH) surge induces oocyte maturation by promoting meiotic resumption and progression to metaphase II (MII). After the reinitiation of meiosis I in these cells, organized nuclear envelope breakdown occurs through a process known as germinal vesicle breakdown (GVBD), after which chromosome condensation, spindle formation, and first polar body (PB) extrusion occur. Oocytes then enter meiosis II only to arrest at MII until fertilization occurs.

The orchestration of oocyte maturation upon gonadotropin stimulation is tightly regulated through bidirectional signaling between oocytes and somatic cells (Hsieh et al., 2007; Conti et al., 2012). The accumulation of cyclic AMP (cAMP) generated through GPR3/12 signaling suppresses maturation-promoting factor expression and thereby promotes oocyte arrest at meiotic prophase I (Conti et al., 2012). The knockout of *Gpr3* interferes with this arrest, resulting in premature oocyte meiosis and a consequent reduction of the oocyte reserve. The absence of GPR3 expression in aging mice leads to markedly reduced fertility as evidenced by an increase in the observed numbers of non-developing embryos following spontaneous ovulation and the large quantities of fragmented oocytes detected following the induction of superovulation (Ledent et al., 2005). The production of follicle-stimulating hormone (FSH) prior to the LH surge served to additionally promote mural granulosa cells (mGCs) to secrete natriuretic peptide precursor type C (CNP/NPPC), which regulates cumulus cell-mediated cyclic GMP (cGMP) production to maintain meiotic arrest in oocytes (Zhang et al., 2010). By entering these cells through gap junctions, cGMP inhibits the activity of the cAMP-degrading enzyme PDE3A in oocytes, thereby furthering meiotic arrest (Richard et al., 2001; Vaccari et al., 2009). The knockout of *Pde3a* can rescue GPR3 deficiency-related abnormal meiotic arrest and premature follicular failure (Vaccari et al., 2008).

An LH surge that occurs proximal to the time of birth can suppress the expression of NPPC/NPR2, thus disrupting the synthesis of cGMP (Sun et al., 2009). LH can also induce the production of key epidermal growth factor (EGF)-like growth factors by mGCs including amphiregulin, beta-cellulin, and epiregulin, which in turn activate cumulus cell EGF receptor signaling and promote the degradation of cGMP in response to the activation of PDE5 and the inactivation of NPR2. These regulatory changes facilitate subsequent oocyte maturation. LH additionally activates ERK1/2 signaling to prevent GC proliferation while inducing GC luteinization (Park et al., 2004). While abnormal meiotic recovery typically results in female infertility (Supplementary Table 1), few studies regarding the role of LH regulation in this process have been conducted using knockout mouse models, highlighting promising directions for future research.

## Telomere Attachment and Movement

Telomere attachment to the nuclear envelope (NE) is a relatively conserved meiotic process. Chromosomal movement during meiotic prophase I, homologous chromosome pairing, synapsis, and recombination all depend on appropriate telomere attachment, which is therefore regulated by multiple telomere binding protein complexes (Zickler and Kleckner, 2016). A shelterin complex covers the chromatin telomeres of most eukaryotic cells (Palm and de Lange, 2008), protecting these structures from degradation, which would trigger irreversible checkpoint activation, senescence, and/or apoptotic death (Palm and de Lange, 2008). The primary protein components of the shelterin complex include TRF1, TRF2, TIN2, POT1, TPP1, and RAP1 (Palm and de Lange, 2008). Of these, TRF1/2 serve as the core shelterin complex proteins. Conditional TRF1 deletion in spermatocytes has been shown to result in a failure of telomere attachment and fusion between telomeres (Wang et al., 2018). Reductions in TRF2 expression can similarly drive abnormal telomere attachment (Hua et al., 2019). In contrast, *Rap1* knockout does not adversely impact telomere attachment or bouquet formation (Scherthan et al., 2011). Further research is necessary to determine whether the other protein components of the shelterin complex play an important role in telomere anchoring during meiosis.

In addition to the shelterin complex, the TERB1-TERB2- MAJIN protein complex is important for the establishment of a second physical linkage for telomere attachment to the NE (Shibuya et al., 2014, 2015). Mutated of these three proteins impairs telomere attachment to the NE and causes infertility in both male and female mice (Shibuya et al., 2014, 2015). TERB1 serves as a molecular scaffold, interacting with both TERB2 as well as the shelterin complex protein TRF1 (Shibuya et al., 2014, 2015; Long et al., 2017). MAJIN is predicted to be a transmembrane protein that localizes to the inner NE surface where it can bind to the SUN Domain protein (Shibuya et al., 2015). The SUN Domain protein, together with the KASH Domain protein, in turn form the linker of nucleoskeleton and cytoskeleton (LINC) complex (Ding et al., 2007), which provides sites for telomere anchoring at the NE and permits NE linkage to the cytoplasm in order to facilitate the force generation necessary to achieve chromosomal movement (Hiraoka and Dernburg, 2009; Tapley and Starr, 2013). By interacting with one another, these proteins generate a robust telomere anchoring complex. Cyclin-dependent kinase 2 (Cdk2) is also known to be important for telomere binding to the inner NE during meiosis (Viera et al., 2015), as is the atypical Cdk activator Speedy A/RingoA, which rapidly induces G2/M progression in oocytes (Mikolcevic et al., 2016; Tu et al., 2017). However, further research will be necessary to understand the precise mechanisms whereby these kinases regulate this stage of meiotic progression.

FBXO47 is an F-box protein that regulates the telomeric shelterin complex necessary for telomere-NE attachment during meiotic prophase I. FBXO47 binds to TRF2 and thereby localizes to telomeres anchored to the nuclear membrane, thereby suppressing TRF2 ubiquitination and degradation. The expression of FBXO47 is testis-specific, suggesting that it plays no role in regulating meiotic prophase I in oocytes and that other compensatory proteins may play a similar role in this context (Hua et al., 2019). Ubiquitination during telomere attachment and movement also differ between the sexes as a consequence of this sex-specific FBXO47 expression pattern.

Overall, patterns of telomere attachment and movement are largely similar in males and females, but data pertaining to FBXO47 suggest that further research into sexual dimorphism during this stage of the meiotic process is warranted.

## DNA Double-Strand Break and Repair

**FIGURE 2:**
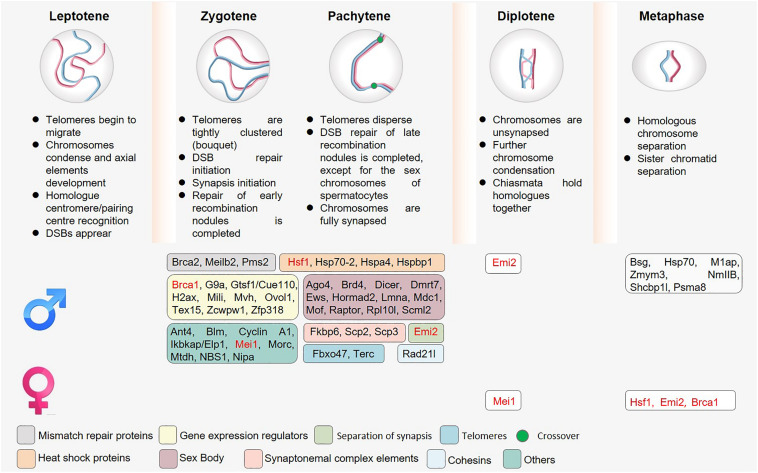
The roles of sex-specific regulatory proteins in meiosis. Owing to overlap in the timing of telomere attachment and movement, double-strand break (DSB) repair, and MSCI, the knockout of proteins associated with these meiotic processes often results in multiple complex phenotypes. Spermatocytes have additionally evolved to express many different sex-specific proteins (shown in boxes). Proteins marked in red indicate the presence of a meiotic phenotype after knockout in both spermatocytes and oocytes, although these phenotypes are not consistent in these two cell types.

DSB formation and DSB repair are key processes that occur during prophase I over the course of 7–8 or 4–5 days in murine spermatocytes and oocytes, respectively (Baudat et al., 2013). Such DSB repair depends upon the initial alignment and recombination of homologous chromosomes, which are governed by homologous DNA recombination and by the synaptonemal complex (SC). Many prior articles have already discussed meiotic recombination in detail, and this topic will thus not be discussed at length in the present review (Zickler and Kleckner, 1999; Roig and Keeney, 2008; Lichten and de Massy, 2011; Baudat et al., 2013). Interestingly, significant sexual dimorphism has been reported with respect to the regulation of chromatin organization, recombination, and meiotic defect tolerance in male and female germ cells (Figure 2). Complete meiotic failure typically arises in male mice harboring mutations that interfere with synapsis or recombination such that cells are unable to progress past the zygotene or pachytene stages, thereby causing spermatocyte apoptosis and consequent infertility. In contrast, these same mutations can result in a spectrum of phenotypes in females ranging from decreased fertility to infertility, ovarian dysgenesis, or embryo loss, likely as a consequence of aneuploidy.

A wide array of proteins serve as essential regulators of DSB formation and repair in the context of meiosis, including SPO11, which is specifically expressed during prophase I and is thought to exert type II DNA topoisomerase-like activity to form DSBs (Keeney et al., 1997). These DSB sites then undergo further processing, potentially through the activity of other regulatory proteins, to generate overhangs that serve as substrates for the RECA homolog single-stranded binding proteins DMC1 and RAD51, which in turn facilitate strand invasion and the formation of double Holliday junctions (Masson and West, 2001). This leads to the interaction of homologous chromosomes. The RAD51 paralog XRCC2 has also been identified as a key driver of RAD51-mediated homologous recombination (Chun et al., 2013). In both humans and mice, a recessive mutation (c.41T > C/p.Leu14Pro) in XRCC2 results in meiotic arrest, azoospermia, and infertility. Notably, however, studies of *Xrcc2^L14P/rm L14P^* mice revealed that while these meiotic and fertility defects were universal in male mice, they affected only half of *Xrcc2^L14P/rm L14P^* female mice, with the remainder of these animals instead exhibiting reduced reproductive capacity (Yang et al., 2018).

After homologous chromosomes interact with one another, a series of structural proteins promote their further stabilization and the initiation of SC formation. The cohesion complex is a ring-shaped structural protein complex that entraps pairs of sister chromatids following replication until the initiation of anaphase at which time the separase-mediated cleavage of the α-kleisin subunits RAD21 or REC8 (during mitosis and meiosis, respectively) results in the opening of the ring structure. This complex serves as an essential recombination scaffold, and is critical for appropriate recombination, synapsis, and chiasmata formation during meiosis. The α-kleisin RAD21L is highly conserved even in non-mammalian species, and is able to interact with the cohesion subunits SMC1, SMC3, and STAG3. The knockout of *Rad21l* in male mice results in defective synapsis during prophase I, in turn causing zygotene arrest, complete azoospermia, and consequent infertility. In contrast, *Rad21l* -deficient females suffer from an age-dependent loss of fertility. SYCE1 and SYCE3 also connect lateral elements via SYCP1 at zygonema, after which TEX12 and SYCE2 loading promotes SC propagation (Fraune et al., 2012). The SC component protein FKBP6 is exclusively expressed in males such that the knockout of this protein impairs spermatocyte meiosis and causes zygotene-like stage arrest in these cells, whereas oocytes are unaffected by the loss of its expression (Crackower et al., 2003; Noguchi et al., 2008).

Yeast model assays have revealed that following strand invasion by one end, strand displacement occurs followed by D-loop formation and invading 3′-end elongation. The other end is then able to interact with the D-loop or the elongated invading strand in respective crossover and non-crossover pathways. PMS2 is a DNA mismatch repair gene homolog, and stabilizing MLH1 levels prior to its critical crossing over function with MLH3. When PMS2 is deleted, complete spermatocyte arrest is similarly observed without any corresponding impact on the development of oocytes (Baker et al., 1995).

A wide range of other DSB–related proteins have been shown to exhibit sexually dimorphic functionality in mammals. For example, TEX15 is an essential regulator of chromosomal synapsis and meiotic recombination, but the knockout of this protein only results in meiotic arrest in male mice without having a corresponding effect on females (Yang et al., 2008). The F–box–like NIPA protein is an E3 ligase and SCF complex member that results in male sterility in conditional knockout models as a consequence of spermatogenesis arrest during meiotic prophase due to improper synapsis, disrupted DSB repair, and abnormal SC formation. In contrast, female *Nipa^–/–^* mice remain fertile albeit with a ∼50% reduction in litter size (Illert et al., 2012). Meiosis-related sexual dimorphism also extends to epigenetic regulatory processes. For example, the H3K4me3-responsive ZCWPW1 protein is necessary for the progression of prophase I in male mice whereas it is dispensable in the context of female murine gametogenesis. *Zcwpw1* knockout in male mice results in the complete disruption of synapsis, causing meiotic arrest at the zygotene/pachytene stage, impaired DSP repair, crossover failure, and infertility. Knockout of ZCWPW1 in oocytes, in contrast, only partially slows the progression of meiotic prophase I, and *Zcwpw1^–/–^* female mice retail normal fertility through mid-adulthood (Li et al., 2019). As the loss of many proteins can result in multi-faceted impacts on meiotic processes, it is important to note that it can be challenging to clearly distinguish between the key regulators of these processes. As such, additional in-depth molecular analyses of these putative regulatory proteins and their targets will be essential to fully appreciate their roles in the context of gametogenesis.

## Meiotic Metaphase

Following the completion of diploid meiotic recombination, germ cells enter a state of transient diakinesis after which spermatocytes/oocytes enter meiotic metaphase to complete the process of meiotic division. Chromosomal alignment at the metaphase plate and subsequent segregation during the initial round of meiotic division are essential to ensure successful meiosis. Several proteins exhibit sexual dimorphism during metaphase. For example, ZMYM3 is a member of the MYM-type zinc finger protein family and a component of an LSD1-containing transcriptional repressor complex. Spermatogenesis in *Zmym3*-knockout mice was arrested during the metaphase of the first meiotic division (MI). ZMYM3 knockout resulted in elevated numbers of apoptotic germ cells and MI spermatocytes that are positive for BUB3, which is a key factor associated with the spindle assembly checkpoint (SAC). In contrast, female *Zmym3*-knockout mice remain fertile (Hu et al., 2017).

The SAC serves as the central regulator of appropriate chromosomal segregation. In a previously published review, Lane et al. have discussed the sexual dimorphism of the meiotic SAC (Lane and Kauppi, 2019), highlighting multiple important proteins that have been studied in the context of metaphase in spermatocytes or/and oocytes. Lower levels of some SAC proteins and more SAC permissiveness in females may result in a higher incidence of meiotic aneuploidy in oocytes relative to spermatocytes.

After the separation of homologous chromosomes, spermatocytes undergo a second round of meiotic division, resulting in the production of four spermatids. However, after the first polar body (PB) is extruded into the space under the zona pellucida, oocytes arrest at MII until fertilization, at which time the second phase of meiotic division is triggered and the extrusion of the second PB occurs. Owing to these phases of regulated meiotic arrest, oocytes are never truly haploid given that their second meiotic division occurs only following fusion with a male germ cell harboring a haploid genome (Bolcun-Filas and Handel, 2018). Several phosphorylation signaling pathway proteins have been found to play important roles in maintaining MII phase arrest in oocytes. For example, MOS is an upstream activator of mitogen-activated protein kinase (MAPK) and, in mouse oocytes, and is responsible for MII arrest (Colledge et al., 1994; Hashimoto et al., 1994). The extracellular regulated kinase-1 and -2 (ERK1/2) cascade plays pivotal roles in regulating oocyte meiotic cell cycle progression. Ovulated *Erk1/2*-knockout oocytes exhibited poorly-assembled MII spindles, spontaneous PB 2 release, and were arrested at metaphase III (MIII). In addition, ERK1/2 deletion prevented male pronuclear formation after fertilization, resulting in female infertility (Zhang et al., 2015). *Ppp2r1a* encodes the scaffold subunit Aalpha of protein phosphatase 2A (PP2A), which is an important and ubiquitously expressed serine-threonine phosphatase family member. Depletion of PP2A-Aalpha facilitates germinal vesicle breakdown, causing elongation of the MII spindle and precocious separation of sister chromatids (Hu et al., 2014). As spermatocytes do not have a MII arrest, these proteins play a specific role only in oocytes.

## Summary

Meiosis is a tightly conserved mammalian biological process that is essential to the generation of male and female gametes, but a high degree of sexual dimorphism exists in the specific regulation of such gametogenesis. Clear differences in the regulation and characteristics of meiotic onset and duration are observed between the sexes (Figure 1). In addition, spermatocytes and oocytes regulate conserved processes through distinct mechanisms, and further exhibit differences in functional processes including unequal follicular cytoplasm differentiation and MSCI that ultimately give rise to these respective gametes (Figure 2). Studying specific proteins using knockout models offers a robust means of exploring their roles in the context of meiotic regulation, and the advent of novel gene-editing technologies has facilitated the identification of a wide array of sexually dimorphic phenotypes exhibited by particular knockout mice.

In this review, we have surveyed over 200 proteins related to meiosis and highlighted those that are functionally sexually dimorphic. However, there are still many other proteins the functions of which have only been reported in a single sex. As meiosis is a sexually dimorphic process, further study of these sex-related differences is warranted. Of the 200+ proteins discovered in this context to date, the molecular functions of many remain to be fully clarified. For example, some of the proteins that cause meiotic arrest in the zygotene and pachytene phases may lead to abnormalities in SC, repair, and telomere attachment. However, differentiation between causes and effects remains challenging. As protein profiling techniques continue to rapidly develop, further screening for protein-protein interactions, small molecule metabolites, and the construction of molecular interaction networks will be beneficial to our understanding of the molecular mechanisms governing meiosis.

By analyzing these sexually dimorphic proteins, we found that a number of spermatocyte-specific proteins are involved in the regulation of gene expression, including histone acetylation mediators, methylation mediators, and transcriptional enhancers/inhibitors. Interestingly, after these genes are mutated, spermatocytes primarily undergo arrest in the early pachytene phase. During the zygotene to pachytene transition, an autosomal transition from transcriptional inhibition to activation occurs. The specific gene expression regulatory proteins involved in the regulation of meiotic processes in spermatocytes suggest that there are distinct and complex gene expression regulatory mechanisms engaged in spermatocytes and oocytes. In addition, it is important to note that the spermatocyte-specific piRNA proteins and associated piRNA/mRNA modifications represent a further dimension regulating spermatocyte gene expression. Despite these advances, defining the purpose underlying observed mechanisms of mammalian germline sexual dimorphism remains challenging. Recent studies have described increasingly intricate technologies allowing for analyses of the regulation of gene expression, such as high-throughput/resolution chromosome conformation capture (Hi-C), Assay for Targeting Accessible-Chromatin with high-throughout sequencing (ATAC-seq), and Full-Length Isoform Sequencing. Additional comprehensive studies leveraging these technologies will aid in more thoroughly elucidating the mechanisms governing meiosis.

## Author Contributions

RH prepared figures and tables, authored draft of the manuscript, and approved the final draft. ML reviewed, revised draft of the manuscript, and approved the final draft. Both authors contributed to the article and approved the submitted version.

## Conflict of Interest

The authors declare that the research was conducted in the absence of any commercial or financial relationships that could be construed as a potential conflict of interest.

## References

[B1] Anderson E. Baltus A. Roepers-Gajadien H. Hassold T. De Rooij D. Van Pelt A. (2008). Stra8 and its inducer, retinoic acid, regulate meiotic initiation in both spermatogenesis and oogenesis in mice. *Proc. Natl. Acad. Sci. USA* 105 14976–14980. 10.1073/pnas.0807297105 18799751 PMC2542382

[B2] Baker S. Bronner C. Zhang L. Plug A. Robatzek M. Warren G. (1995). Male mice defective in the DNA mismatch repair gene PMS2 exhibit abnormal chromosome synapsis in meiosis. *Cell* 82 309–319. 10.1016/0092-8674(95)90318-6 7628019

[B3] Baltus A. Menke D. Hu Y. Goodheart M. Carpenter A. De Rooij D. (2006). In germ cells of mouse embryonic ovaries, the decision to enter meiosis precedes premeiotic DNA replication. *Nat. Genet.* 38 1430–1434. 10.1038/ng1919 17115059

[B4] Barzel A. Kupiec M. (2008). Finding a match: how do homologous sequences get together for recombination? *Nat. Rev. Genet.* 9 27–37. 10.1038/nrg2224 18040271

[B5] Baudat F. Imai Y. De Massy B. (2013). Meiotic recombination in mammals: localization and regulation. *Nat. Rev. Genet.* 14 794–806. 10.1038/nrg3573 24136506

[B6] Bhalla N. Dernburg A. (2008). Prelude to a division. *Annu. Rev. Cell Dev. Biol.* 24 397–424. 18597662 10.1146/annurev.cellbio.23.090506.123245 PMC4435778

[B7] Bolcun-Filas E. Handel M. A. (2018). Meiosis: the chromosomal foundation of reproduction. *Biol. Reprod.* 99 112–126. 10.1093/biolre/ioy021 29385397

[B8] Bowles J. Knight D. Smith C. Wilhelm D. Richman J. Mamiya S. (2006). Retinoid signaling determines germ cell fate in mice. *Science* 312 596–600. 10.1126/science.1125691 16574820

[B9] Buonomo S. Clyne R. Fuchs J. Loidl J. Uhlmann F. Nasmyth K. (2000). Disjunction of homologous chromosomes in meiosis i depends on proteolytic cleavage of the meiotic cohesin Rec8 by separin. *Cell* 103 387–398. 10.1016/s0092-8674(00)00131-8 11081626

[B10] Cahoon C. Hawley R. (2016). Regulating the construction and demolition of the synaptonemal complex. *Nat. Struct. Mol. Biol.* 23 369–377. 10.1038/nsmb.3208 27142324

[B11] Chen H. Welling M. Bloch D. Muñoz J. Mientjes E. Chen X. (2014). DAZL limits pluripotency, differentiation, and apoptosis in developing primordial germ cells. *Stem Cell Rep.* 3 892–904. 10.1016/j.stemcr.2014.09.003 25418731 PMC4235140

[B12] Chun J. Buechelmaier E. Powell S. (2013). Rad51 paralog complexes BCDX2 and CX3 act at different stages in the BRCA1-BRCA2-dependent homologous recombination pathway. *Mol. Cell. Biol.* 33 387–395. 10.1128/mcb.00465-12 23149936 PMC3554112

[B13] Colledge W. Carlton M. Udy G. Evans M. (1994). Disruption of c-mos causes parthenogenetic development of unfertilized mouse eggs. *Nature* 370 65–68. 10.1038/370065a0 8015609

[B14] Conti M. Hsieh M. Zamah A. Oh J. (2012). Novel signaling mechanisms in the ovary during oocyte maturation and ovulation. *Mol. Cell. Endocrinol.* 356 65–73. 10.1016/j.mce.2011.11.002 22101318 PMC4104635

[B15] Crackower M. Kolas N. Noguchi J. Sarao R. Kikuchi K. Kaneko H. (2003). Essential role of Fkbp6 in male fertility and homologous chromosome pairing in meiosis. *Science* 300 1291–1295. 10.1126/science.1083022 12764197 PMC2882960

[B16] Ding X. Xu R. Yu J. Xu T. Zhuang Y. Han M. (2007). SUN1 is required for telomere attachment to nuclear envelope and gametogenesis in mice. *Dev. Cell* 12 863–872. 10.1016/j.devcel.2007.03.018 17543860

[B17] Endo T. Freinkman E. De Rooij D. Page D. (2017). Periodic production of retinoic acid by meiotic and somatic cells coordinates four transitions in mouse spermatogenesis. *Proc. Natl. Acad. Sci. USA* 114 E10132–E10141. 29109271 10.1073/pnas.1710837114 PMC5703301

[B18] Fraune J. Schramm S. Alsheimer M. Benavente R. (2012). The mammalian synaptonemal complex: protein components, assembly and role in meiotic recombination. *Exp. Cell Res.* 318 1340–1346. 10.1016/j.yexcr.2012.02.018 22394509

[B19] Gerton J. Hawley R. (2005). Homologous chromosome interactions in meiosis: diversity amidst conservation. *Nat. Rev. Genet.* 6 477–487. 10.1038/nrg1614 15931171

[B20] Gondos B. Byskov A. Hansen J. (1996). Regulation of the onset of meiosis in the developing testis. *Ann. Clin. Lab. Sci.* 26 421–425. 8879359

[B21] Handel M. A. Schimenti J. C. (2010). Genetics of mammalian meiosis: regulation, dynamics and impact on fertility. *Nat. Rev. Genet.* 11 124–136. 10.1038/nrg2723 20051984

[B22] Handel M. Eppig J. (1998). Sexual dimorphism in the regulation of mammalian meiosis. *Curr. Top. Dev. Biol.* 37 333–358. 10.1016/s0070-2153(08)60179-9 9352191

[B23] Hashimoto N. Watanabe N. Furuta Y. Tamemoto H. Sagata N. Yokoyama M. (1994). Parthenogenetic activation of oocytes in c-mos-deficient mice. *Nature* 370 68–71. 10.1038/370068a0 8015610

[B24] Hiraoka Y. Dernburg A. F. (2009). The SUN rises on meiotic chromosome dynamics. *Dev. Cell* 17 598–605. 10.1016/j.devcel.2009.10.014 19922865

[B25] Hsieh M. Lee D. Panigone S. Horner K. Chen R. Theologis A. (2007). Luteinizing hormone-dependent activation of the epidermal growth factor network is essential for ovulation. *Mol. Cell. Biol.* 27 1914–1924. 10.1128/mcb.01919-06 17194751 PMC1820474

[B26] Hu M. Wang Z. Jiang Z. Qi S. Huang L. Liang Q. (2014). Scaffold subunit Aalpha of PP2A is essential for female meiosis and fertility in mice. *Biol. Reprod.* 9:19. 10.1095/biolreprod.114.120220 24899574

[B27] Hu X. Shen B. Liao S. Ning Y. Ma L. Chen J. (2017). Gene knockout of Zmym3 in mice arrests spermatogenesis at meiotic metaphase with defects in spindle assembly checkpoint. *Cell Death Dis.* 8:e2910. 10.1038/cddis.2017.228 28661483 PMC5520888

[B28] Hua R. Wei H. Liu C. Zhang Y. Liu S. Guo Y. (2019). FBXO47 regulates telomere-inner nuclear envelope integration by stabilizing TRF2 during meiosis. *Nucleic Acids Res.* 47 11755–11770. 31724724 10.1093/nar/gkz992 PMC7145685

[B29] Hunt P. Hassold T. (2002). Sex matters in meiosis. *Science* 296 2181–2183. 10.1126/science.1071907 12077403

[B30] Illert A. Kawaguchi H. Antinozzi C. Bassermann F. Quintanilla-Martinez L. Von Klitzing C. (2012). Targeted inactivation of nuclear interaction partner of ALK disrupts meiotic prophase. *Development* 139 2523–2534. 10.1242/dev.073072 22696294 PMC3383228

[B31] Keeney S. Giroux C. Kleckner N. (1997). Meiosis-specific DNA double-strand breaks are catalyzed by Spo11, a member of a widely conserved protein family. *Cell* 88 375–384. 10.1016/s0092-8674(00)81876-0 9039264

[B32] Keeney S. Lange J. Mohibullah N. (2014). Self-organization of meiotic recombination initiation: general principles and molecular pathways. *Annu. Rev. Genet.* 48 187–214. 10.1146/annurev-genet-120213-092304 25421598 PMC4291115

[B33] Kojima M. De Rooij D. Page D. (2019). Amplification of a broad transcriptional program by a common factor triggers the meiotic cell cycle in mice. *eLife* 8:e43738 10.7554/eLife.43738 PMC6392498 30810530

[B34] Koubova J. Menke D. Zhou Q. Capel B. Griswold M. Page D. (2006). Retinoic acid regulates sex-specific timing of meiotic initiation in mice. *Proc. Natl. Acad. Sci. USA* 103 2474–2479. 10.1073/pnas.0510813103 16461896 PMC1413806

[B35] Kudo N. Wassmann K. Anger M. Schuh M. Wirth K. Xu H. (2006). Resolution of chiasmata in oocytes requires separase-mediated proteolysis. *Cell* 126 135–146. 10.1016/j.cell.2006.05.033 16839882

[B36] Lam I. Keeney S. (2014). Mechanism and regulation of meiotic recombination initiation. *Cold Spring Harb. Perspect. Biol.* 7:a016634. 10.1101/cshperspect.a016634 25324213 PMC4292169

[B37] Lane S. Kauppi L. (2019). Meiotic spindle assembly checkpoint and aneuploidy in males versus females. *Cell. Mol. Life Sci.* 76 1135–1150. 10.1007/s00018-018-2986-6 30564841 PMC6513798

[B38] Ledent C. Demeestere I. Blum D. Petermans J. Hämäläinen T. Smits G. (2005). Premature ovarian aging in mice deficient for Gpr3. *Proc. Natl. Acad. Sci. USA* 102 8922–8926. 10.1073/pnas.0503840102 15956199 PMC1150279

[B39] Li M. Huang T. Li M. Zhang C. Yu X. Yin Y. (2019). The histone modification reader ZCWPW1 is required for meiosis prophase I in male but not in female mice. *Sci. Adv.* 5:eaax1101. 10.1126/sciadv.aax1101 31453335 PMC6693912

[B40] Lichten M. de Massy B. (2011). The impressionistic landscape of meiotic recombination. *Cell* 147 267–270. 10.1016/j.cell.2011.09.038 22000007 PMC3263351

[B41] Lin Y. Gill M. Koubova J. Page D. (2008). Germ cell-intrinsic and -extrinsic factors govern meiotic initiation in mouse embryos. *Science* 322 1685–1687. 10.1126/science.1166340 19074348

[B42] Liu W. Wang F. Xu Q. Shi J. Zhang X. Lu X. (2017). BCAS2 is involved in alternative mRNA splicing in spermatogonia and the transition to meiosis. *Nat. Commun.* 8:14182. 10.1038/ncomms14182 PMC5290162 28128212

[B43] Long J. Huang C. Chen Y. Zhang Y. Shi S. Wu L. (2017). Telomeric TERB1-TRF1 interaction is crucial for male meiosis. *Nat. Struct. Mol. Biol.* 24 1073–1080. 10.1038/nsmb.3496 29083416 PMC6108177

[B44] MacLean G. Li H. Metzger D. Chambon P. Petkovich M. (2007). Apoptotic extinction of germ cells in testes of Cyp26b1 knockout mice. *Endocrinology* 148 4560–4567. 10.1210/en.2007-0492 17584971

[B45] Masson J. West S. (2001). The Rad51 and Dmc1 recombinases: a non-identical twin relationship. *Trends Biochem. Sci.* 26 131–136. 10.1016/s0968-0004(00)01742-4 11166572

[B46] Matson C. Murphy M. Griswold M. Yoshida S. Bardwell V. Zarkower D. (2010). The mammalian doublesex homolog DMRT1 is a transcriptional gatekeeper that controls the mitosis versus meiosis decision in male germ cells. *Dev. Cell* 19 612–624. 10.1016/j.devcel.2010.09.010 20951351 PMC2996490

[B47] Mikolcevic P. Isoda M. Shibuya H. Del Barco Barrantes I. Igea A. Suja J. (2016). Essential role of the Cdk2 activator RingoA in meiotic telomere tethering to the nuclear envelope. *Nat. Commun.* 7:11084. 10.1038/ncomms11084 PMC4820962 27025256

[B48] Moor R. Warnes G. (1979). Regulation of meiosis in mammalian oocytes. *Br. Med. Bull.* 35 99–103. 10.1093/oxfordjournals.bmb.a071578 387169

[B49] Morelli M. Cohen P. (2005). Not all germ cells are created equal: aspects of sexual dimorphism in mammalian meiosis. *Reproduction* 130 761–781. 10.1530/rep.1.00865 16322537

[B50] Nicholls P. Schorle H. Naqvi S. Hu Y. Fan Y. Carmell M. (2019). Mammalian germ cells are determined after PGC colonization of the nascent gonad. *Proc. Natl. Acad. Sci. USA* 116 25677–25687. 10.1073/pnas.1910733116 31754036 PMC6925976

[B51] Noguchi J. Ozawa M. Nakai M. Somfai T. Kikuchi K. Kaneko H. (2008). Affected homologous chromosome pairing and phosphorylation of testis specific histone, H2AX, in male meiosis under FKBP6 deficiency. *J. Reprod. Dev.* 54 203–207. 10.1262/jrd.19158 18408354

[B52] Palm W. de Lange T. (2008). How shelterin protects mammalian telomeres. *Annu. Rev. Genet.* 42 301–334. 10.1146/annurev.genet.41.110306.130350 18680434

[B53] Park J. Su Y. Ariga M. Law E. Jin S. Conti M. (2004). EGF-like growth factors as mediators of LH action in the ovulatory follicle. *Science* 303 682–684. 10.1126/science.1092463 14726596

[B54] Richard F. Tsafriri A. Conti M. (2001). Role of phosphodiesterase type 3A in rat oocyte maturation. *Biol. Reprod.* 65 1444–1451. 10.1095/biolreprod65.5.1444 11673261

[B55] Roig I. Keeney S. (2008). Probing meiotic recombination decisions. *Dev. Cell* 15 331–332. 10.1016/j.devcel.2008.08.009 18804427

[B56] Sakuno T. Tanaka K. Hauf S. Watanabe Y. (2011). Repositioning of aurora B promoted by chiasmata ensures sister chromatid mono-orientation in meiosis I. *Dev. Cell* 21 534–545. 10.1016/j.devcel.2011.08.012 21920317

[B57] Scherthan H. Sfeir A. De Lange T. (2011). Rap1-independent telomere attachment and bouquet formation in mammalian meiosis. *Chromosoma* 120 151–157. 10.1007/s00412-010-0295-4 20927532 PMC3132479

[B58] Sedlmeier E. M. Brunner S. Much D. Pagel P. Ulbrich S. E. Meyer H. H. (2014). Human placental transcriptome shows sexually dimorphic gene expression and responsiveness to maternal dietary n-3 long-chain polyunsaturated fatty acid intervention during pregnancy. *BMC Genomics* 15:941. 10.1186/1471-2164-15-941 25348288 PMC4232618

[B59] Seligman J. Page D. (1998). The Dazh gene is expressed in male and female embryonic gonads before germ cell sex differentiation. *Biochem. Biophys. Res. Commun.* 245 878–882. 10.1006/bbrc.1998.8530 9588208

[B60] Shibuya H. Hernandez-Hernandez A. Morimoto A. Negishi L. Hoog C. Watanabe Y. (2015). MAJIN links telomeric DNA to the nuclear membrane by exchanging telomere cap. *Cell* 163 1252–1266. 10.1016/j.cell.2015.10.030 26548954

[B61] Shibuya H. Ishiguro K. Watanabe Y. (2014). The TRF1-binding protein TERB1 promotes chromosome movement and telomere rigidity in meiosis. *Nat. Cell Biol.* 16 145–156. 10.1038/ncb2896 24413433

[B62] Sun Q. Miao Y. Schatten H. (2009). Towards a new understanding on the regulation of mammalian oocyte meiosis resumption. *Cell Cycle* 8 2741–2747. 10.4161/cc.8.17.9471 19717979

[B63] Tapley E. Starr D. (2013). Connecting the nucleus to the cytoskeleton by SUN-KASH bridges across the nuclear envelope. *Curr. Opin. Cell Biol.* 25 57–62. 10.1016/j.ceb.2012.10.014 23149102 PMC3578026

[B64] Tu Z. Bayazit M. Liu H. Zhang J. Busayavalasa K. Risal S. (2017). Speedy A-Cdk2 binding mediates initial telomere-nuclear envelope attachment during meiotic prophase I independent of Cdk2 activation. *Proc. Natl. Acad. Sci. USA* 114 592–597. 10.1073/pnas.1618465114 28031483 PMC5255603

[B65] Turner J. (2015). Meiotic silencing in mammals. *Annu. Rev. Genet.* 49 395–412. 10.1146/annurev-genet-112414-055145 26631513

[B66] Vaccari S. Horner K. Mehlmann L. Conti M. (2008). Generation of mouse oocytes defective in cAMP synthesis and degradation: endogenous cyclic AMP is essential for meiotic arrest. *Dev. Biol.* 316 124–134. 10.1016/j.ydbio.2008.01.018 18280465 PMC2755085

[B67] Vaccari S. Weeks J. Hsieh M. Menniti F. Conti M. (2009). Cyclic GMP signaling is involved in the luteinizing hormone-dependent meiotic maturation of mouse oocytes. *Biol. Reprod.* 81 595–604. 10.1095/biolreprod.109.077768 19474061 PMC2731981

[B68] van Pelt A. de Rooij D. (1991). Retinoic acid is able to reinitiate spermatogenesis in vitamin a-deficient rats and high replicate doses support the full development of spermatogenic cells. *Endocrinology* 128 697–704. 10.1210/endo-128-2-697 1989855

[B69] Viera A. Alsheimer M. Gómez R. Berenguer I. Ortega S. Symonds C. (2015). CDK2 regulates nuclear envelope protein dynamics and telomere attachment in mouse meiotic prophase. *J. Cell Sci.* 128 88–99. 10.1242/jcs.154922 25380821

[B70] Wang L. Tu Z. Liu C. Liu H. Kaldis P. Chen Z. (2018). Dual roles of TRF1 in tethering telomeres to the nuclear envelope and protecting them from fusion during meiosis. *Cell Death. Differ.* 25 1174–1188. 10.1038/s41418-017-0037-8 29311622 PMC5988695

[B71] Watanabe Y. (2012). Geometry and force behind kinetochore orientation: lessons from meiosis. *Nat. Rev. Mol. Cell Biol.* 13 370–382. 10.1038/nrm3349 22588367

[B72] Yang F. Eckardt S. Leu N. Mclaughlin K. Wang P. (2008). Mouse TEX15 is essential for DNA double-strand break repair and chromosomal synapsis during male meiosis. *J. Cell Biol.* 180 673–679. 10.1083/jcb.200709057 18283110 PMC2265566

[B73] Yang Y. Guo J. Dai L. Zhu Y. Hu H. Tan L. (2018). XRCC2 mutation causes meiotic arrest, azoospermia and infertility. *J. Med. Genet.* 55 628–636. 10.1136/jmedgenet-2017-105145 30042186 PMC6119352

[B74] Zhang M. Su Y. Sugiura K. Xia G. Eppig J. (2010). Granulosa cell ligand NPPC and its receptor NPR2 maintain meiotic arrest in mouse oocytes. *Science* 330 366–369. 10.1126/science.1193573 20947764 PMC3056542

[B75] Zhang T. Zarkower D. (2017). DMRT proteins and coordination of mammalian spermatogenesis. *Stem Cell Res.* 24 195–202. 10.1016/j.scr.2017.07.026 28774758 PMC5634931

[B76] Zhang T. Murphy M. Gearhart M. Bardwell V. Zarkower D. (2014). The mammalian Doublesex homolog DMRT6 coordinates the transition between mitotic and meiotic developmental programs during spermatogenesis. *Development* 141 3662–3671. 10.1242/dev.113936 25249458 PMC4197572

[B77] Zhang T. Oatley J. Bardwell V. Zarkower D. (2016). DMRT1 is required for mouse spermatogonial stem cell maintenance and replenishment. *PLoS Genet.* 12:e1006293. 10.1371/journal.pgen.1006293 27583450 PMC5008761

[B78] Zhang Y. Liu X. Ji S. Sha Q. Zhang J. Fan H. (2015). ERK1/2 activities are dispensable for oocyte growth but are required for meiotic maturation and pronuclear formation in mouse. *J. Genet. Genomics* 42 477–485. 10.1016/j.jgg.2015.07.004 26408092

[B79] Zickler D. Kleckner N. (1999). Meiotic chromosomes: integrating structure and function. *Annu. Rev. Genet.* 33 603–754. 10.1146/annurev.genet.33.1.603 10690419

[B80] Zickler D. Kleckner N. (2016). A few of our favorite things: pairing, the bouquet, crossover interference and evolution of meiosis. *Semin. Cell Dev. Biol.* 54 135–148. 10.1016/j.semcdb.2016.02.024 26927691 PMC4867269

